# “Big chunks of blank memory”: complex trauma and dissociative body memory

**DOI:** 10.1007/s11019-025-10274-7

**Published:** 2025-05-03

**Authors:** Jake Dorothy

**Affiliations:** Department of Philosophy, University of York, York, UK

**Keywords:** Complex trauma, Phenomenological psychopathology, Body memory, Dissociation, Amnesia, CPTSD

## Abstract

Research into traumatic memory has focused heavily upon re-experiencing symptoms (e.g. flashbacks). Features predominantly associated with complex trauma, such as gaps in the recollection of traumatic events, remain comparatively underexplored. In this article, I draw on the testimonies of survivors of complex trauma who participated in a survey informed by Phenomenologically Grounded Qualitative Research (Køster and Fernandez in Phenomenol Cogn Sci 22:149, 2023). I provide a phenomenological account of how survivors often experience memory blanks as inchoately disturbing, despite being unable to recount ‘missing’ events. Although challenging and equivocal, the notion of body memory offers one way of articulating this phenomenon. Specifically, I suggest that the troubling feelings accompanying perceived gaps in recollection arise alongside a form of non-conceptual body memory, which, lacking in propositional content, fails to be meaningfully contextualised. Drawing on the literature on body memory, dissociation, and Husserl’s (Collected works. Kluwer, Dordrecht, 1991 [1893–1917]) internal time consciousness, I distinguish this as dissociative body memory and describe two central, non-exhaustive, features: (1) habitual dissociation, and (2) protentive salience. What is taken to be a gap in traumatic memory is in fact only a partial gap, involving a kind of pre-reflective remembering that is not recognised as such. Dissociative body memory additionally prevents the narrative integration required for minimising these perceived gaps, leading to an ongoing sense of foreboding concerning one’s past. This has significant clinical implications, highlighting that what survivors experience as forgotten must not be disregarded. At the theoretical level, the phenomenon may be a hitherto unrecognised characteristic of complex posttraumatic stress disorder and related conditions.

## Introduction

The role of memory in posttraumatic experience has long attracted attention. Since the inception of research on psychological trauma in the early twentieth century, it has been remarked that “…certain happenings would leave indelible and distressing memories—memories to which the sufferer was continually returning, and by which he was tormented day and night” (Janet 1919/25, p. 589, cited in van der Kolk and van der Hart 1991). Indeed, much emphasis has been placed upon posttraumatic symptoms associated with re-experiencing in the form of flashbacks and nightmares, such that they comprise part of the diagnostic criteria for both posttraumatic stress disorder (PTSD) and complex posttraumatic stress disorder (CPTSD) (World Health Organization [WHO] 2019). However, many survivors^1^ of complex trauma—chronic, typically developmental, interpersonal trauma—also face significant *amnesia*: gaps in recollection for traumatic events, and, in some cases, posttraumatic daily life. Somewhat surprisingly, this remains poorly understood.

Complex trauma is causally implicated in a range of psychiatric conditions. According to the classifications of the 11th revision of the International Classification of Diseases (ICD-11 [WHO] 2019) these include CPTSD, borderline personality disorder (BPD) and dissociative identity disorder (DID) (Dorahy et al. 2014; Stepp et al. 2016). Although psychiatric diagnostic categories are not clear-cut (Hyman 2021) and this is not an exhaustive list, these conditions share dissociation as a core symptom (Hyland et al. 2020; Lyssenko et al. 2018). While it has been rightfully pointed out that dissociation lacks conceptual clarity (Berrios 2018; Černis et al. 2018; van der Hart et al. 2006), it is broadly accepted that dissociation involves discontinuity across or disconnection between a range of aspects of consciousness, including memory (WHO 2019). Given that dissociation is distressing and associated with self-harm and suicide attempts (Foote et al. 2008; Şar 2011), it is important that memory blanks are not considered less problematic than re-experiencing. Phenomenological psychopathology promises to be helpful in addressing this lacuna, with its potential to articulate the structures at stake in posttraumatic amnesia.

While amnesia following complex trauma is heterogenous, certain experiences appear prevalent within survivor testimonies. In this article, I focus on one such experience: namely, the way in which gaps in trauma memory are encountered as inchoately threatening, despite the object of the threat resisting description. In other words, even though survivors are unable to recount the event that is ‘missing’, feelings of unease and disturbance arise when seeking to approach or confront memory blanks, as if something predatory lurks within and might coalesce from the gap. In the words of one survivor, approaching trauma memory for which experiential recall is profoundly impaired results in feeling “nauseous, shaky and sensitive” or, alternatively, “almost incorporeal” [Participant 33]. Notably, it is not the gap itself that is the object of threat, but what lies beyond it: akin to a closed door signifying something frightening only in light of what might potentially lie behind it. In investigating this phenomenon, I draw upon survivor testimony from my own qualitative research and the existing media sources which helped form my background research.

Although distressing forgetfulness is not exclusive to conditions associated with complex trauma, it is evidently pronounced and prevalent in these cases. Failure in episodic recollection is an intrinsic feature of human memory, an ability that predictably deteriorates with age (Rowlands 2017). However, the vast majority of our forgetting is readily accommodated and a relatively inconspicuous feature of life. While there will always be some everyday exceptions to this (e.g. following excessive alcohol consumption), as well as in other pathological conditions (e.g. early-stage dementia), it is likely that the lived experience of trauma-related amnesia is troubling in a distinct way. For one thing, it includes cases in which a survivor’s memory blanks are so profound that it appears there are no conceptual grounds for believing trauma to have occurred; yet there exists a tangible sense of something missing, accompanied by a sense of dread and foreboding. Given the complexity of memory, such gaps are equivocal and multifactorial. Their explication, however, will require accommodating not only those who have some knowledge of a traumatic past (i.e. those who retain partial recollection or possess corroborating evidence), but those for whom this is less obvious.

The following phenomenological account, drawing upon the notion of body memory (Casey 2000; Fuchs 2012, 2017), proposes one way in which this might be achieved. Specifically, I suggest that what is experienced as a distressing gap in trauma memory in fact only a partial gap, wherein a conspicuous sense of ‘something missing’ arises alongside a form of non-conceptual body memory. Lacking in propositional content, this resists meaningful contextualisation as the object of one’s distress remains unarticulated. As such, survivors’ pre-reflective embodied recollection acts as a kind of remembering that is not clearly identified as such. Given the role of dissociation in complex trauma and its posttraumatic symptomatology, I propose that dissociation is central to its development, and thus distinguish it as *dissociative body memory*. I describe two central, non-exhaustive, aspects: (i) habitual dissociation, and (ii) protentive salience. Where the former emphasises dissociation as a habituated response that over time arises pre-reflectively, and is a kind of remembering that emphasises absent propositional content, the latter seeks to account for the exacerbation of the inchoate threat that accompanies this. Here, I draw on Edmund Husserl’s (1991 [1893–1917]) internal time consciousness to propose that dissociative body memory involves an unusual protentive salience, whereby conscious awareness of the past and present becomes diminished in favour of a tacit expectation of future harm.

I begin by outlining complex trauma and dissociation, before focusing on survivor testimonies from a phenomenologically-informed survey that report memory blanks as disturbing. I then describe the notion of body memory and set out my account of dissociative body memory. To conclude, I address a potential objection concerning similarities between body memory and affective responses to traumatic reminders, and briefly consider clinical and theoretical implications.

## Complex trauma and memory

### Complex trauma, psychopathology, and dissociation

Many people will experience at least one potentially traumatising event in their life. For a significant minority, the negative repercussions are consistent with a diagnosis of PTSD or CPTSD. While PTSD tends to be associated with a single traumatising event of either interpersonal or non-interpersonal nature (e.g. a one-off assault, life-threatening acute illness, or natural disaster), CPTSD typically follows prolonged interpersonal trauma, otherwise known as complex trauma (Barrett and Fish 2014). This is often developmental (e.g. neglect or physical, emotional and sexual abuse in childhood), but also occurs in response to domestic violence, torture, combat and displacement.

While CPTSD shares symptoms with PTSD (e.g. re-experiencing, avoidance of reminders and hypervigilance), it additionally involves severe problems in affective regulation, persistent negative self-beliefs associated with guilt and shame, and relational difficulties (WHO 2019). CPTSD involves greater functional impairment (Murphy et al. 2021) and appears less responsive to existing therapies (Karatzias et al. 2019). While various factors are behind this, one concerns the greater severity of dissociation in CPTSD (Hyland et al. 2020). Dissociation is similarly recognised as a significant problem in other conditions associated with complex trauma (e.g. BPD and DID; WHO 2019), with which survivors may be separately diagnosed or express comorbidly. Regardless of an individual’s precise diagnosis, therefore, a link exists between complex trauma and dissociative phenomena.

While there remains disagreement about the theoretical construct of dissociation, a range of experiences have been consistently described as dissociative by prominent clinicians and researchers such as Herman (2004 [1992]), Bessel van der Kolk et al. (e.g. 1996), and van der Hart et al. (2006). These experiences have been more recently thematised by Černis et al. (2018) as subjective feelings of unreality (including depersonalisation and derealisation); feeling emotionally disconnected; memory blanks; ‘zoning out’; and having a vivid internal world (including intrusions and hallucinations). Many authors agree that amnesia is a feature of traumatic dissociation (e.g.; Herman 2004 [1992]; Loewenstein 2014; Nijenhuis et al. 2001; van der Hart and Brom 2000; van der Hart et al. 2006), with DID being prototypical in this regard. While the severity of memory blanks in posttraumatic daily life varies considerably, gaps in memory for traumatic events themselves are commonly reported (Briere and Conte 1993; Cameron 2000; van der Hart et al. 2005).

### Methodology

This article draws upon the written testimonies of individuals with CPTSD who describe gaps in traumatic memory. These data were gathered as part of a broader online survey exploring the ways in which complex trauma affects experiences of selfhood. Participants were recruited via social media and university mailing lists, as well as third-sector mental health organisations and university campuses, and were eligible if they were aged eighteen or over and self-identified as having experienced at least one traumatic event. Ethical approval was granted by the University of York’s Arts and Humanities Ethics Committee.

78 of a total of 113 participants met the criteria for CPTSD according to the International Trauma Questionnaire ([ITQ]; Cloitre et al. 2018), with trauma histories spanning emotional, physical or sexual abuse in childhood, domestic violence, sexual assault, bullying, being a witness to violence, traumatic loss and medical trauma.

Participants completed an open-ended fifteen-item questionnaire whose design was informed by Phenomenologically Grounded Qualitative Research methodology (PGQR) as described by Køster and Fernandez (2023). Questionnaire methodology has been central to recent large-scale projects within phenomenological psychopathology (e.g. Ratcliffe 2014, 2023) and is particularly valuable in achieving bigger sample sizes. PGQR emphasises the conceptual front-loading of questionnaire items in order to establish a specific focus upon phenomenological structures; specifically, selfhood in relation to embodiment, temporality and intersubjectivity, in which memory is centrally implicated. Extensive prior research on pre-reflective and reflective self-experience, as well the symptoms of dissociation and traumatic memory, guided the construction of the questionnaire. Two questions focused explicitly on memory, one of which invited participants to describe associated experiences of embodiment (Q6: “Does your memory of traumatic events seem different to your memory of non-traumatic events?” and Q7: “How does your body feel when you have memories of the trauma?”). See Table 1 for a complete list of open-ended questionnaire items.

**Table 1:** Open-Ended questionnaire items

1. How would you describe your daily life? This might include your daily activities, any positive features of your life, any problems you regularly experience, and how satisfied you are with your life overall. If you remember a time before the trauma, you may wish to compare your daily life now with how it was before the trauma occurred.
2. How do you find being with people you are familiar with (e.g. your partner, friends, family, colleagues, or therapist)? This might include how being around familiar people makes you feel, your ability to feel connected to familiar people, and your ability to trust.
3. How do you find being around people you are unfamiliar with? This might include how being around unfamiliar people makes you feel, your ability to communicate with unfamiliar people, and your ability to trust.
4. How would you describe yourself? This might include the attributes you consider yourself to have, how you feel about yourself, and whether you ever switch between holding very different views about yourself.
5. Do you experience any difficulties with your emotions? This might include how emotions feel to you, or the ways in which you respond to your emotions.
6. Does your memory for traumatic events seem different to your memory for other, non-traumatic life events? If so, please elaborate on the ways in which they seem different.
7. How does your body feel when you have memories of the trauma?
8. Does your sense of sight, hearing, smell, touch or taste sometimes seem different to how you ordinarily experience it? If so, please elaborate on how you experience this, in what kind of situations, and its impact.
9. Does your experience of time passing sometimes seem different to how you ordinarily experience it? If so, please elaborate on how you experience this, in what kind of situations, and its impact.
10. Have you ever had the experience of seeing yourself from the outside, or a sense of being outside your body? If so, please describe this experience and the situation/s it occurred in.
11. Have you ever had visions that nobody else could see, or heard a voice/voices that nobody else could hear? If so, please describe this experience and the situation/s it occurred in.
12. Do you sometimes feel as though your thoughts, feelings or behaviours are not your own? If so, please elaborate.
13. Do you feel as if a part of yourself has been lost as a result of trauma? If so, please elaborate.
14. How do you think or feel about the future?
15. What do you believe to be the most significant ways in which trauma has affected you?

While the overarching framework of the study was phenomenological and performed with a philosophical aim, initial analysis took the form of coding loosely derived from Reflexive Thematic Analysis (Braun and Clarke 2006, 2021). This enabled identification of prominent themes among participants, highlighting the full range of survivor experiences and their frequencies before being evaluated for theoretical significance. Notably, amnesia was directly mentioned 24 times, with other potentially related experiences frequently referred to (e.g. memory being fuzzy, like a film, or not one’s own). This comprised a sizeable subset of participants, and given that traumatic memory blanks are underresearched in contrast to vivid posttraumatic recollection, there was a strong theoretical case for this article’s focus.

Of those participants who experienced memory blanks (and those survivors whose accounts I have quoted from various media sources) the foci of habitual dissociation and protentive salience were among the most common across the empirical material. They are also theoretically compatible with empirical research concerning dissociation, as will become clear. Crucially, this investigation into the phenomenology of traumatic memory blanks helps characterise the experiential world of survivors beyond re-experiencing, emphasising concomitant loss and disconnection.

### Threatening memory blanks

In posttraumatic memory associated with PTSD, re-experiencing symptoms typically predominate. Flashbacks of affective and perceptual aspects of the traumatising event are characterised as richly detailed, immutable and unforgettable. While they may involve interrupted chronology and be difficult to narrativize (Herman 2004 [1992]), insofar as the content recalled, the balance tends towards heightened recollection. Memory blanks in PTSD may be considered akin to brief spells of signal disruption on an otherwise exceptionally clear radio bandwidth. There may be occasional missing or scrambled details, but not so much as to detract understanding of the overall message. Here, the torment arises from involuntary remembering.

For those with CPTSD and related conditions, however, there may exist a greater proportion of memory blanks relative to that recalled. This varies significantly between individuals: some may remember some, but not all, of their trauma history (e.g. one of several perpetrators, half of the times one was assaulted, or a handful of details from a period spanning years), whilst others report no recollection but a conspicuous sense of something missing and undefined lurking in their past. Similarly, some report amnesia for trauma for years until a striking reminder or corroborating evidence emerges. For survivors of complex trauma, therefore, gaps in recollection are analogous to prolonged radio signal disturbances that sufficiently disrupt understanding of the message. It is evident that *some* kind of message is being transmitted, but the content remains obscure and difficult to fathom. Indeed, many describe their memories as blurry, of impaired quality that are difficult to access: “it’s hazy, like fragmented bits and then blankness in between” (Participant 35). As one participant points out, details remain elusive: “it seems blurred into one, and also speeded up at times. I cannot remember exact details or how I felt at the time if asked to remember” (Participant 101).

Some survivors describe an ambiguous sense of both contextually understanding their trauma histories *and* experiencing overt absence concerning the details. As one participant notes:

The traumatic events that happened to me were mainly pushed away in my brain, but I get a small memory, like where I don’t remember exactly what happens but I understand the basics of it all. (Participant 8)

Such experiences are undoubtedly complex and open to a range of interpretations. The challenge is in explaining how one might have a memory involving overarching knowledge of one’s trauma (e.g. “I was abused”) whilst simultaneously having little to no recollection of what this entailed (i.e. when, where, with whom or how the abuse took place). One interpretation is to draw a distinction between the recollection of one’s trauma in semantic terms and the recollection of experiential content. While in PTSD recollection is likely to be rich in experiential content—knowing *how* an event occurred, in overwhelming detail—following complex trauma, survivors may only appear to know *that* a given trauma took place.

This distinction between knowing ‘how’ and knowing ‘that’ has generally been taken to be a difference in kind of memory, namely episodic and semantic (Tulving 1972). According to Tulving, semantic memory is a store of general knowledge that can be drawn upon *without* reference to the events comprising its formation, while episodic memory involves a kind of ‘mental time-travel’ that *does* involves recollecting the events that formed the memory. In terms of autobiographical memory, therefore, semantic memory involves ‘remembering *that*’ about oneself, whilst episodic memory involves remembering the autobiographical situation itself. Thus whereas semantic autobiographical memories are attributed to a person by way of a *‘that’-*clause (e.g. “I remember that I was tortured”), episodic autobiographical memories are attributed by way of an incomplete *dependent* clause, involving not a complete sentence describing an autobiographical event, but an active recollection of *being tortured*. While semantic memories thus express a proposition, episodic memories do not, and the object of semantic memory is different in kind to the object of an episodic memory.

The extent to which these types of memory are themselves different in kind is debated. Episodic autobiographical memories are obviously rich in experiential content; semantic autobiographical memories may lack this altogether or possibly retain partial experiential colouring. However, even among those who consider the difference between semantic and episodic autobiographical memories to be one of degree and not kind, there is thought to be something distinctive about episodic autobiographical memories. Mark Rowlands (2017) has argued that the *sine qua non* of episodic autobiographical memories is that they involve the recollection of an event as “one formerly witnessed, orchestrated, or otherwise experienced” *by oneself* (p. 50); a particularly experiential mode of presentation of the memory. Where survivors lack this mode of presentation of past traumatic events—which minimally involves one experiencing the episode as one formerly witnessed—innumerable aspects of the experience are lost to them, resulting in the peculiar sense that one knows what happened despite also drawing conspicuous blanks^2^:

The memory of the traumatic event is more so a name for that event [rather] than anything recollective as a normal everyday memory would be. I’ve never tried to think of specifics of what happened or think about it more as I doubt that would be enjoyable. (Participant 45)

I don’t remember a lot of things, and memories of bad times kind of just look the same to our normal memories. I know this is because we don’t have access to the experiential memory (or it wasn’t stored), so we’re just reconstructing a scene from the data. They kinda just look like weird photographs, often third person. Kind of frozen, maybe flickering between different elements of a scene. We don’t have a lot of specifically traumatising memories that we acknowledge or know were traumatising. (Participant 25)

Possessing episodic memories of historic trauma is far from desirable, given the distress it understandably triggers. Yet amnesia is itself troubling. Not only are there practical concerns if ongoing episodes interfere with everyday functioning, but gaps in trauma memory may be experienced as particularly disquieting; something discomfitingly ill-defined, giving rise to inchoate fear and doubt. In her documentary project *Paper Birds*, Brooke (2022) presents a detailed account of how a disturbing, conspicuous sense of a ‘forgotten’ trauma in her past led to uncontrolled, somatic manifestations of distress despite the object of the memory resisting articulation:

It was a Tweet or something, so something completely insignificant, but when I read it something in my body just clicked and suddenly I remembered something happening in my childhood…I could remember that something had happened but I couldn’t remember what that something was…I waited for it to go away, but it wouldn’t and it just felt like there was this box in my head, that there was something there but I just didn’t know what was inside of it….

She describes asking her mother whether anything ‘ever happen[ed]’ to her as a child:

…as I said the words “the box” out loud…I felt immediately like I was pulled backwards, and that I was sucked back into my body, and I’d never experienced anything like that before. And I just couldn’t…my hands just started shaking really violently, and I started crying and hyperventilating and I didn’t know what was going on, I just lost complete bodily autonomy. And the only thing I could say was “Mom, I don’t know what’s happening, Mom, I don’t know what’s happening”…she tried to get me to calm down, but then she couldn’t… I remember that moment being like “oh shit, I was right, something did happen.” (Brooke 2022).

In her testimony, the nagging sense of discomfort towards the memory blank seems to be increasingly tangibly felt upon confronting it—the “box”– head on. By acknowledging or approaching what is lost, an unknown predatory threat might emerge. That a box is chosen as a metaphor to describe an inaccessible memory supports the notion that what is feared is not the gap itself, but what lurks within it (i.e. the contents of the box). This sense of something ‘missing’ and troubling arises in numerous survivor testimonies. For some, this is characterised as a loss, while for others it is encountered as something vague and obscure, dark and best kept at a distance:

Forgetting. I wonder if this sounds like a good thing. Maybe it does? After all, if you forget aspects of abuse, it won’t bother you, right? Wrong. All I really know is that my whole childhood has stretches of absolutely nothing at all. Blankness, regardless of what might have happened in that space…I call these blank passages of time ‘empty rooms’. That’s what they feel and look like when I think of them: grey-scale, entirely bare, deserted, with wind blowing through them. They are all over the place. And I can’t help but feel them as losses. (Debney 2022)

My memories of traumatic events seem to be grey in colour and dark. Some are hard to remember. (Participant 67)

It is fuzzy, not clear, partial, I don’t want to remember any of it. (Participant 103)

Indeed, for some, what has been lost is more frightening than what is remembered:

I have moments of extreme clarity of what happened as well as big chunks of blank memory with the traumatic events. I have parts I’ll never forget as well as some I’ll never remember, and the parts I can’t remember are the parts I’m more scared of. (Participant 12)

The experience of memory blanks as disturbing is intuitive in cases where survivors have partial recollection of their trauma. Holding conceptual awareness of a traumatic past is a *prima facie* reason for fearing perceived gaps; one is justified in expecting the worst. However, this construes survivors’ experience of threat as deriving purely from cognitive processes. This view is undermined by the way in which individuals reporting no recollection until later in life typically initially describe discomfort in certain situations which, unbeknownst to them, are reminiscent of their trauma. Others sense ‘something missing’ in their pasts that is distressing yet apparently unexplained, accompanied by somatic symptoms that become troubling enough to seek psychotherapeutic support. Reaching out to professionals in this way is testament to the degree of confusion surrounding threat responses which become distressing owing to a growing unease around their lack of explanation. In such cases, it is the embodied, uncontextualised fear of an explicit unknown that prompts survivors to seek answers:

My PTSD [sic] of an event from fifteen years ago was triggered last year, and this manifested itself into night terrors that I couldn’t remember, and physical reactions of feeling unsafe and in danger, especially when it came to intimate situations. Through working with a psychologist, I am gradually remembering my trauma. (Participant 71)

One participant described such profound disconnection from trauma memories recalled at the at the age of seventeen that she doubted their reliability before finding corroborating evidence, illustrating the extent to which memory blanks may predominate in CPTSD:

It all seems a bit blurry compared to normal memories, there are also gaps in the timeline. I’ve had trauma-induced amnesia in the past, I’ve forgotten the memory of childhood abuse for years till they all came back when I was seventeen. It’s the kind of memories that at first you don’t believe, it all seemed unbelievable till I found proof. I don’t think a person would normally doubt their memory. (Participant 72)

The experience of trauma-related memory blanks as profound and imbued with danger is evidently prevalent in survivors of complex trauma. This may potentially be characteristic of conditions such as CPTSD, BPD and DID, despite being currently underemphasised in diagnostic manuals and clinical assessments. While there are other circumstances in which this phenomenon might reasonably occur (e.g. in substance misuse issues or early-stage dementia), such dis-ease around lack of recollection is, in general, not a defining feature of peoples’ lives. Following complex trauma, however, living with such gaps may comprise a great deal of suffering. Rather than being accommodated without distress, they are explicitly felt, acting as additional barriers to the integration of traumatic memory. As Corey (2024) notes, “it is difficult to acknowledge there are aspects of my own life of which I have no awareness. It’s scary.”

## Body memory

I have described how survivors may variously retain *some* episodic autobiographical memory (i.e. parts of their trauma history, to differing degrees) or none whatsoever, recounting it in semantic terms that fail to capture the details of what occurred. In extreme cases, some may lack any semantic knowledge whatsoever. What is minimally shared, however, is the loss of at least *some* episodic content and experiential richness informing survivors of *how* they were harmed.

That this loss is experienced as conspicuous and distressing suggests that the loss cannot be solely conceived of in terms of absence. It is not merely the lack of episodic memory at stake, but also the presence of certain affective and embodied attitudes which characterise the gaps as a lingering threat. As one survivor notes, with the guidance of her therapist, the body is central in how this is experienced:

I only remember parts of my trauma. Sometimes I have feelings in my body I don’t understand and can’t place, but I find them distressing. My therapist says this is a body memory. My daily life is a blur but it doesn’t haunt me like the past does. (Participant 97)

While this testimony involves a psychological conceptual import, it does highlight the extent to which the body is implicated in distressing feelings that characterise a “haunt[ing]” past. If embodiment is plausibly implicated in how memory blanks are experienced, one possible way of elucidating them pertains to the conception of body memory.

Within the phenomenological tradition, the term body memory denotes the acquired dispositions, skills and habits that constitute an individual’s present experiences and capacities. A form of habitual memory, it is akin to the kind of remembering that cognitive science tends to classify as procedural or implicit (Schachter 1987), in contrast to declarative memory that includes both semantic and episodic memory.

According to Bergson (2007 [1896]), bodily habits accrue from repeated movements, postures and gestures, determining our attitudes and automatic reactions towards objects perceived in the present. Merleau-Ponty (2012 [1945]) further emphasises these tacit responses by associating the habitual body (*corps habituel*) with the lived body: the body’s pre-reflective capacity for seeing, touching and moving which is established in all ordinary experience despite rarely featuring as an object of conscious awareness itself. He describes the habitual body not as representing the past, but as enacting it by means of “operative intentionality” (Merleau-Ponty 2012 [1945], p. 122). This is famously depicted in his illustration of a typist and organist: while learning to type or play requires conscious and deliberate effort to become accustomed to the keys, repetition eventually permits a tacit know-how and ability to perform the task with spontaneity. Such fluent performance does not entail knowledge involving explicit recollection of the movements required, but a practical attunement developed through experience that is tantamount to habitual knowledge residing within the lived body.

In this way, body memory guides our everyday being-in-the-world, facilitating interaction with objects and other persons. It is not only formed by learning processes, but also interpersonal rituals, cultural norms, values, and styles of expression (Tewes and Fuchs 2018). In spite of its embodied features, therefore, it cannot be reduced to sensorimotor functions. It corresponds with some enactivist approaches to cognitive science which explore the possibility that remembering might be better conceived as a type of interaction, such as re-enactment (e.g. Hutto and Myin 2017). Indeed, working from within the phenomenological tradition, Casey (2000) notes that body memory re-enacts the past without the need to represent it. Thus if we are to call body memory a form of memory (and following Casey, I will), it refers to the implicit, embodied remembering that re-enacts habitual experience and facilitates interaction across self, other and world.

The vast range of bodily dispositions one acquires over the course of a lifetime has led to different types of body memory being distinguished (e.g. Fuchs 2012, 2017). While some allow for smooth performance and flexible interaction with the people and the world, others involve distinctly maladaptive disruption and rigidity. Both Casey (2000) and Fuchs (2012) have identified traumatic or trauma body memory, which functions comparatively maladaptively. Casey notes that traumatic memories in general may be various in character, for example memory of painful thoughts, but that traumatic *body* memory “*arise[s] from and bear[s] on* one’s own lived body in moments of duress” (p. 154, emphasis added). As such, there is a clear distinction between, e.g. painful episodic recollections of trauma that trigger shame, and traumatic body memory in which the body is centrally implicated as both giving rise to the memory and undergoing its effects^3^.

On Casey’s conception, traumatic body memory particularizes distinct aspects of the body such that the lived body is experienced as fragmented, failing to align smoothly with the surrounding world, separating individuals from it and disrupting spontaneous action. Fuchs (2012) instead considers trauma to leave the most indelible impression the body, arguing that repetitive harm is sedimented within the lived body and reactualised in moments corresponding to one’s trauma history. These include the intense reactions involved in re-experiencing, as well as subtle predispositions to threat perception. He notes that these may arise even when details of the trauma are withdrawn from conscious recollection.

On these accounts, memory may be non-conceptual and lacking in propositional content, yet retain pre-reflectively embodied affective, physiological, perceptual, social, and temporal features. Accordingly, individuals with a history of trauma may fail to conceptually grasp what certain bodily sensations signify, yet live daily with pre-reflective reminders of the horrors which have historically befallen them. In complex trauma, therefore, what is understood as amnesia may relate only the loss of conceptual episodic (and potentially semantic) autobiographical memory; habitual, non-conceptual memory remains intact.

However, Casey’s and Fuchs’ accounts require some adapting if they are to accommodate the way in which memory blanks are experienced in complex trauma. While they provide a convincing explanation for how those with PTSD undergo disruption to ordinary functioning and reactualisation of past harm (e.g. in pain flashbacks), these experiences appear phenomenologically distinct from amnesia. The experience of perceived memory blanks is not a vivid re-experiencing or re-enactment, but a feeling of absence alongside inchoate threat and disturbance. Two aspects in particular thus require elucidation. Firstly, how gaps in memory, and, more specifically, a sense of there being something missing, is made explicit. And secondly, how these gaps are accompanied by inexplicable distress. To address this, I distinguish a type of trauma body memory as *dissociative body memory* and illustrate two central features in turn: habitual dissociation, and protentive salience.

## Dissociative body memory

### Habitual dissociation

According to Casey, traumatic body memory has a proclivity to particularise parts of the body, emphasising them such that the lived body’s ordinarily fluid capacities become disrupted. In the case of CPTSD and related conditions, however, embodied responses may appear more subtly; not a direct re-living of pain in a part of the body that causes its objectification, but a diffuse discomfort or sense of disconnection from one’s body. Although Fuchs notes that trauma body memory can be apparent in both vivid re-experiencing and in more subtle responses that are withdrawn from conscious recollection, his account lacks an explanation for the vast disparity between these types of trauma body memory.

If harms from trauma are similarly sedimented in the lived body over time, what then influences the extent to which their reactualisation is vivid and accompanied by conceptual recollection, or, conversely, hazy and shrouded in disconnection and loss? CPTSD, BPD and DID arise from *chronic* trauma—that is, an increased number of episodes in which to establish embodied responses to harm (e.g. vivid pain or startle response)—yet involve greater memory blanks. I suggest that one way of understanding this is to distinguish such trauma memory as involving *habitual dissociation*. This also gives rise to the tangibility of survivors’ gaps in trauma memory: their sense that there is something ‘missing’ at all.

Increased levels of dissociation in complex trauma lend strong evidence to the idea that broader dissociative phenomena might inform the structure and dominance of non-conceptual body memory. Notably, peri-traumatic dissociation is particularly associated with complex trauma (van der Hart et al. 2008). This is generally conceived as a protective mechanism; an attempt to psychologically distance oneself from otherwise inescapable harm (Herman 2004 [1992]). In this context, dissociation refers to a wide range of phenomena, many of which implicate alterations in embodied experience. This includes feelings of depersonalisation or unreality (e.g. feeling as though one doesn’t exist), alterations in sensory perception (e.g. differences in the experience of sight, sound, or touch), and the experience of observing oneself in the third person (e.g. autoscopic hallucinations or out-of-body experiences) (Černis et al. 2018; van der Hart et al. 2006). Such experiences are generally accompanied by a sense of powerlessness, arising involuntarily and corresponding with an inability to move or speak that emphasise the extent to which peri-traumatic dissociation overcomes ordinary bodily capacity. Two survivors describe this in their experiences of abuse:

The first time [I had an out-of-body experience] was the first time I was raped when I was sixteen, and I remember looking at myself in the bed (because I was trapped and couldn’t move, partially because I was still half asleep) and I just remember that feeling of being frozen and it was awful and it was like I was straining to get up and get out but I just couldn’t. (Participant 52)

Within the traumatic experience itself, floating up to the ceiling, zoning out, seeing myself from the outside. Within an abusive marriage, often feeling like I am watching a film about myself, not connected to my body, feeling like I am disappearing. (Participant 27)

Peri-traumatic dissociation is a significant predictor of posttraumatic dissociation (van der Hart et al. 2008). Posttraumatic dissociation can also become a more generalised response to comparatively minor stressors (van der Hart et al. 2006), suggesting that these phenomena can develop habitually. In this way, those with histories of complex trauma may continue to display a tacit, embodied disposition towards dissociating, a response whose development can be clearly traced back to recurrent incidents of harm in which safety and bodily integrity was at risk.

If body memory is to be understood as “the totality of implicit dispositions of perception and behaviour mediated by the body and sedimented in the course of earlier experience” (Fuchs 2012, p. 86), then the ongoing posttraumatic dissociation that follows repeated peri-traumatic dissociation may reasonably comprise a form of body memory. Feelings of unreality, sensory alterations, and alterations in ordinary perspectival orientation may play a significant role in what are felt to be uncontrollable reactions to approaching both retained episodic recollections of trauma *and* the gaps in one’s past.

What is experienced as a gap is in fact therefore only a *partial* gap; dissociative body memory remains accessible. Put another way, dissociative phenomena *are* what is being remembered in the absence of the propositional content that discloses details of traumatic events. What is being actualised in the present is the embodied disposition of dissociation and its correlating dissociative phenomenon, which can itself be recalled and articulated (albeit to varying degrees). Corresponding with this, though, given its status as a form of non-conceptual memory, is a distinct lack of autobiographical episodic recollection of the very events such dissociation concerns.

On this account, dissociative phenomena comprise a particular kind of pre-reflective recollection—body memory—which, when actualised, makes explicit its lack of meaningful contextualisation. Dissociative body memory itself characterises the sense of conspicuous loss survivors report when unable to remember autobiographical episodic details of their traumatising events. They undergo significant disruptions to ordinary embodied experience, vividly illustrating that something troubling exists; yet the precise nature of this resists articulation. One survivor describes approaching trauma memories, surmising that they are associated with early sexual experiences, but, instead of demonstrating experiential, episodic recall, is overcome with alterations in embodiment:

[Out of body experiences] still occur now when I recall most of the traumas in my life. I also experienced this once very acutely when with friends. It feels generally like a floating or disconnected sense but with the friends was more like literally flying away. This particular occasion had occurred because they were discussing first sexual experiences. (Participant 4)

These alterations in embodiment also described in cases where episodic autobiographical memory is intact, suggesting that dissociative phenomena are key features of memory in complex trauma in general. That is, they characterise underlying, world-orienting body memory in cases where experiential recall is both absent *and* present:

When I try to remember my childhood, for example shameful events I can still remember, I don’t see them from my own perspective but from a third-person perspective (for example I see myself in the classroom together with other pupils). (Participant 20)

For those who experience memory blanks, dissociative responses can be so profound that survivors report having no sense of the even the semantic content, despite conceptual awareness that they have been ‘triggered’ or reminded of a traumatising subject. Here, it would appear that non-conceptual body memory expresses acquired habitual dissociation to such an extent that survivors remain unable to ‘know’ what their traumatic memories are about at all, while the memory blanks remain conspicuous:

There are some traumatic memories I have which are pulled to the surface as a result of other people mentioning their own trauma. These are *memories I don’t realise I have*. And when this occurs my brain disassociates and I switch off entirely. (Participant 67, emphasis added)

The kinds of alteration in embodiment undergone in peri-traumatic dissociation closely correspond to those involved in attempts to recount one’s traumatic past. As shown in the testimonies above, survivors approach these gaps to find themselves “flying away” or “switching off entirely”, a defensive response that discloses one’s ongoing orientation to annihilation. In severe circumstances, where survivors report no recollection of trauma whatsoever, dissociation may be so profound that even non-conceptual body memory is distanced from.

Importantly, these alterations in embodiment are not analogous to observer memories (Nigro and Neisser 1983), a common phenomenon whereby individuals’ episodic recollection for certain autobiographical events, over time, becomes represented from a third-person perspective. In observer memories, episodic memory mutates and takes on a different character of presentation. For example, in recalling oneself playing football and scoring, one ‘sees’ oneself shooting into the goal from an imagined, externalised point of view. Here, the content of the memory may be accurate in that one did indeed score a goal, but its perspective does not maintain fidelity to the original experience. In contrast, in complex trauma, the feeling of “flying away” or witnessing oneself from the ceiling *does* maintain fidelity to one’s experience at the time. Where dissociation arises as an acquired disposition in the form of body memory, it derives from the past repeated embodied interactions as they have actually occurred in one’s life.

Habitual dissociation evidently often forms part of many survivors’ experiential landscapes in a way that is distinct from semantic or episodic recollection. This pre-reflective, non-conceptual body memory importantly lacks propositional content. It is not evident from experiencing the range of involuntarily embodied responses precisely *what* they are about. Even if one grows to recognise that they are more likely to occur in certain situations or develops an understanding of dissociation in psychotherapy, the objects that one’s implicit responses are directed towards resist articulation. Thus survivors may come to understand that their dissociation signifies *something* traumatic, but lack the capacity to understand what that something is. As such, ongoing alterations in embodiment, despite being a form of memory of the past, are unable to be meaningfully contextualised.

An explanatory gap emerges, whereby ordinary bodily experience is disrupted but one simply cannot explain why. This incongruity is, in part, what accounts for survivors’ sense of there being something missing. Having no propositional content with which to furnish distressing embodied experiences with meaning, the presence of dissociative body memory characterises trauma histories as replete with gaps. The perceived memory blanks are not only tangibly felt, but likely to be further emphasised by the distancing effects of dissociation itself: disconnecting from contextualising information as a protective strategy, yet resulting in confusing symptoms which, given their unusual nature, draw attention to themselves. This confusion has the potential to compound the overt discrepancy between body memory and conceptual understanding. Whereas other kinds of body memory, such as the procedural memory involved in dynamic sequences of activity (Fuchs 2012), also have the potential to become separated from conceptual understanding, these are not negatively valanced in the way that trauma memory is. Any loss of conceptual understanding is therefore more likely to remain inexplicit.

To summarise, dissociative body memory involves the tacit reactualisation of habitual dissociation deriving from past traumatic experiences, but its lack of accompanying propositional content renders it uncontextualised. This, as well as the anomalous nature of dissociation itself, leads to a conspicuous sense of something ‘missing’ in one’s past, as what is felt as a gap in memory is only a partial one (i.e. in episodic and sometimes semantic autobiographical memory). This contributes to troubling feelings, as what is ‘missing’ is inexplicable. However, I suggest that an additional aspect of experience is at stake in the distress experienced; namely, temporality.

### Protentive salience

The experience of posttraumatic recollection (or attempted recollection) involves its own temporality. Indeed, PTSD has been described as involving altered temporal experience on account of its re-experiencing symptoms, confining survivors to an endless present in which the trauma is repeated (Mezzalira 2021). In complex trauma, I suggest that the temporality of dissociative body memory influences the inchoate sense of fear attached to memory blanks.

In ordinary pre-reflexive experience, the embodied intentionality of internal time consciousness constitutes the unified temporal horizon of past, present, and future (lived time). According to Husserl (1991 [1893–1917]), embodied selfhood structures lived time such that our acts are always located within the now that divides our retained past and anticipated future. His phenomenology of internal time consciousness considers how the perception of temporal objects is possible given an enduring act of consciousness (e.g. a melody), and how this consciousness unifies itself across time.

Husserl takes the *primal impression* to be the “source-point” of the enduring object (1991 [1893–1917], p. 30). Impressions, being the first of time-constituting phenomena, allow for consciousness of an object (e.g. a single note) that is simultaneous with the current phase of consciousness (i.e. the present moment). On their own, however, these are insufficient, as one would simply experience ever-changing contents in the now without awareness of their departure into the past. For this, *retention* is required, whereby previous phases of consciousness and their intentional content is retained. Thus, in listening to music, it is possible to hear the relation and established continuity of notes as they progress, constructing a melody. The experience of previous notes remains, despite the lack of perceptual content to sustain their presence. Indeed, they diminish in clarity and undergo modification to the extent that they eventually fall away, becoming fainter as they recede into the past. For Husserl, “a retention” captures the notion of the dying away of what is impressionally, and thus bodily, experienced. This modification is a serial process in which retentional contents include their own retentioning: retention “changes into retention of retention and does so continuously. Accordingly, a fixed continuum of retention arises in such a way that each later point is retention for every earlier point” (1991 [1893–1917], p. 31). Thus, an intentional unification of consciousness is maintained across time.

Our sense of time does not merely concern the past and present, however. It also involves anticipation of the future. Husserl’s notion of *protention* is that consciousness has an intentional sense of events yet to come, akin to retention but running in the direction of the future. For example, when one listens to a familiar melody, one is able to anticipate its upcoming notes so that one’s expectations are fulfilled upon hearing them. Indeed, the existence of surprise is enabled by protentive phenomena: were an unexpected change to occur in the melody, one would react with disappointment or discomfort. Husserl notes that, in anticipating the future, we find contents to be interpreted, which result in objective protentions. These also have a mediated intentionality, a serial process opposite to that of retention; “a having-in-advance of a having-in-advance…of a future impression” (Mensch 2021, p. 49). Rather than fading, protentions of a content increase in vividness, the successions either culminating in perceptual content that fulfils expectation, or in a sense of incompleteness at its lack. Given that the content of protention is not always determinate, however, protentive content may also appear as a general sense of an ‘unspecified something’ about to occur.

The lived body’s intentional constitution of impression, retention, and protention thus organises experience into a coherent temporal structure. It also helps structure memory. Ordinarily, in remembering, one is conscious of one’s situatedness in the present and of one’s past in memory: “just as I see being-now in perception and enduring being in the extended perception as it becomes constituted, so I see the past in memory” (Husserl 1991 [1893–1917], p. 36). Primary memory involved in retention, in response to actually present perception, can develop into the reproduction of temporal objects in secondary memory, occurring “independently without being joined to perceptions” (1991 [1893–1917], p. 37). While recollection of the past without immediate primal impressions (e.g. imagining a melody one heard at a concert several weeks ago) is *like* perception and primary memory, its fact of being past is “a given fact, given itself and therefore ‘perceived’” (1991 [1893–1917], p.38). Therefore while recollection itself is presently and originally constituted recollection, secondary memory involves remembering something no longer given as present and available to perception which is accompanied by awareness of it being a representation of the past.

With regards to trauma memory blanks, however, the past is not a consciously “remembered, re-presented past”. It is not the case that one reflectively considers one’s trauma history and that its past status is given as part of the experience of remembering, informing one’s ongoing response to it in the present. Rather, in the case of approaching memory blanks, one passively undergoes dissociative body memory that is not recognised as the past as such, pre-reflectively reactualising dissociation in the present and entailing a primal impression of *being dissociated*. Although this response is based upon repeated episodes of trauma, and in this sense derives from the past, there is an impairment in the degree to which it has been consigned to secondary memory (under the Husserlian conception), while the original threat has long since passed from retention. Accordingly, the lived experience of dissociative body memory cannot be adequately made sense of by way of what has passed. Indeed, retention of dissociative body memory is itself likely to be severely impaired given that swiftly passing from a dissociated state to a non-dissociated state involves a distinct lack of phenomenal recollection:

I stop having any awareness of my senses for a short while. I’m usually unaware of it happening until afterwards. I could be watching a film and all of a sudden I can’t hear or see anything, then I will become aware of it again and can remember events but I don’t remember experiencing them. (Participant 74)

While this self-report suggests that the *primary* function of retention may not be completely lacking, given that there is *some* recollection of the dissociation having taken place, it is plausible that an aspect of retention, possibly its affective modification^4^ (Husserl 2001 [1920–1926]), is affected such that there is a notable diminishment in recalling significant aspects of the experience. Accordingly, the experience of dissociative body memory involves a diminished sense of both past and present, resulting in an unusual emphasis on the sense of *what is to come*. In other words, its protentive structure has altered and is salient relative to inaccessible secondary memory and disconnection from the perceptual reality of the present moment.

I have already established that aspects of secondary memory, which may be considered akin to episodic memory, can be perceived as absent in complex trauma, to varying degrees. Additionally, where dissociative body memory arises, its inability to be meaningfully contextualised prevents it from being consigned to the past. The habitual memory being reactualised fails to unproblematically pass into retention and from there into secondary memory; rather, it is a shock every time, one that defies understanding. In approaching memory blanks, therefore, internal time consciousness of the past is altered, becoming both unavailable and unreliable, with survivors’ capacities to episodically recall historic events and conceptualise them as having already taken place dramatically reduced.

Similarly, the distancing effects of dissociative phenomena impair perceptual awareness of the present moment and, adjacently, temporal awareness of the present. Their anomalous qualities, fostering a sense of disconnection from one’s current self and environment, result in a diminished ability to perceive external stimuli as they are actually taking place. This is apparent in phenomena such as alterations in perspectival orientation and sensory alterations, but also in the discrepancy between being in safe surroundings while experiencing anxiety towards memory blanks. In other words, dissociative body memory arises even when one’s current external situation presents no risk of harm. Despite the present moment conveying no risk, therefore, what is prominently felt *is* a kind of threat, albeit an unspecified one. This suggests that the temporal contents of the primal impression are diminished in comparison to the sense of anticipation: that, in dissociative body memory, survivors of complex trauma undergo a diminished experience of both past and present in favour of an inchoate *expectation* of harm; future, unexplained harms.

Given its theorised role as a protective strategy in response to historic, repeated trauma, it is unsurprising that habitual dissociation involves a significant embodied, pre-reflective expectation of harm. Accordingly, survivors may experience a sense of anticipation of danger with increasing vividness as dissociative body memory is undergone, corresponding to the degree of ‘closeness’ to the missing memory (as depicted in A.C. Brooke’s detailed account, where her distress increased upon specifying the “box” shrouding her past). Acknowledging the mounting feeling of future-oriented risk corresponding to dissociative body memory helps explain how survivors may express extreme anxiety around memory blanks, despite having little conceptual awareness of what they concern. Admittedly, the origin of the threat and fear may also lie with the dissociative experience itself; the dramatic alterations to embodiment which, in their extremity, are a shocking disruption to ordinary experience. However, that survivors’ experiences of dissociation often culminate in surprise, relief, or confusion upon recognizing that harm has not actually come to pass illustrates the salience of the underlying protentive structure in complex trauma memory.

The following testimony depicts how dissociative body memory may be experienced in particular contexts despite diminished secondary memory. Even though this participant experiences gaps in episodic autobiographical memory, she remains predisposed to expect danger in certain given situations. Survivors may not be able to explain why a particular person, object, or place entails any threat, yet there is a sense of impending danger. Given the lack of contextualisation for the memory, this sense of risk may be challenging to describe:

It seems like I’ll have very vivid memories, like if I’m remembering a non-traumatic event, but the memories of the traumatic events will be very distant, blurry, dreamlike, or feel like I’m remembering something I was told rather than something I remember…Now I’ll feel partially separated in a stressful situation like during an argument. I experienced it a lot as a kid, but more completely out of the body. I’ll feel like I was floating behind [my] body and watching myself go through the motion of school or other tasks. (Participant 61)

Dissociative body memory is reactualised in such a way that an expectation of approaching harm is made explicit, in spite of the object of the threat remaining unspecified. While repeated dissociative experiences might help an individual to identify certain contexts as triggers (e.g. environmental or relational), the precise object of the threat is obscured owing to the lack of propositional content. A survivor might learn to recognise that he undergoes an out-of-body experience passing a particular building, yet struggle to properly identify what this specifically *mean*s (i.e. what actually happened to him around that building). This phenomenon indicates that while past and present temporal experience is impaired (i.e. episodic memory fails to furnish understanding of the experience, and embodied alterations reduce awareness of the present), a pre-reflective anticipation of harm predominates.

Husserl notes that protentive phenomena can be unspecified, given that the future is not always possible to predict. On this account, it is therefore possible that a pronounced, yet unspecified, expectation of harm can permeate survivors’ internal time consciousness. That protention remains unspecified (i.e. uncontextualised) despite the anticipation-orientation being so pronounced is likely to worsen distress. In dissociative body memory, survivors are disconnected from both secondary memory and primal impressions, instead oriented towards a projected future threat that arises in confusing and distressing circumstances. Accordingly, memory blanks are characterised by an explicit expectation of something bad, unpleasant, or frightening emerging, even if survivors are unable to fully reason why.

## Conclusion

In this article, I explored how conditions associated with complex trauma can involve gaps in memory which are experienced as inchoately disturbing. To date, this has been poorly understood. Although the phenomenon is heterogenous and equivocal, any account must accommodate the fact that a subset of survivors report distress despite having no initial recollection of their trauma histories whatsoever. I suggested that this might be understood via the notion of body memory, and proposed that a form of non-conceptual dissociative body memory is present despite a lack of corresponding episodic or semantic memory. I identified two non-exhaustive features of dissociative body memory: habitual dissociation, wherein tacit dissociative responses acquired through past trauma are reactualised and their lack of contextualisation made explicit, and protentive salience, where a pre-reflective, embodied orientation towards the anticipation of future harm is emphasised. Together, these explain how memory blanks are experienced as conspicuous and threatening, as opposed to blending unproblematically into the background.

Dissociative body memory is not only a form of remembering, but one which itself emphasises perceived gaps. Its distancing effects and accompanying distress further prevent the integration required to minimise them. In emphasising a sense of foreboding that resists meaningful contextualisation, the narrativisation deemed integral to trauma recovery (Brison 2002; Herman 2004 [1992]) is hard to come by. Psychotherapy for complex trauma might therefore seek to encourage survivors to recognise dissociation as a kind of remembering, rather than solely conceptualising episodic and semantic memories as such. Doing so might help reduce associated difficulties, as the threat of what might emerge from the gap is no longer the primary focus. Treating embodied, dissociative responses as memories in their own right could help divert attention away from the fear of discovering something additional lurking in one’s past.

It is worth noting that there may be variations in the experiences of dissociative body memory which have not yet been fully explored. The majority of participants in this study reported predominantly interpersonal trauma such as abuse, bullying, the sudden loss of a loved one, or witnessing harm to another person. These kind of events and their relation to posttraumatic amnesia may be different to other kinds of trauma that are complicated by additional alterations in consciousness and memory. For example, traumatic events that take place alongside the consumption of substances (particularly dissociative substances, such as ketamine or nitrous oxide) or the experience of being ventilated on an intensive care unit during the height of the Covid-19 pandemic, where many patients described confusion over what was real. Future research may wish to investigate dissociative body memory in relation to such populations.

It might be objected that dissociative responses are simply associated with affective states, rather than constituting any kind of memory. One might suggest, for example, that dissociation in response to something reminiscent of the trauma (e.g. the smell of a particular aftershave) is merely due to challenging emotions directed towards that object (e.g. shame or anger). I do not wish to discount the affective element, particularly given that heightened negative emotional sensitivity is closely connected to dissociation (Černis et al. 2022). However, to suggest that such responses are *solely* due to affectivity towards a stimulus underplays the role of chronic repetition in the development of ongoing dissociation, as well as failing to accommodate its involuntary, pre-reflective qualities and the extent to which posttraumatic dissociation is a disproportionate response to one’s present environmental context.

Moreover, the notion of body memory extends the traditional conceptualisation of memory as explicitly representational, and questions the assumption that the basis of memory is internal to the brain (Froese and Izquierdo 2018). On this conceptualisation, the acquired embodied dispositions that one has learned in relation to and through interaction with the world can be thought of as a kind of remembering. As Malafouris and Koukouti (2018) state, “the way a body remembers its skills is by re-enacting them inside the world using available forms of material culture and not by representing them inside the brain” (p. 158). While this may seem more intuitively applicable to adaptive forms of body memory, such as procedural body memory, it is no less the case for comparatively maladaptive forms such as traumatic body memory. The reactualisation of habitual dissociation in response to a certain environmental or social context can, in this way, be understood as memory and not merely affective response. As Casey (2000) points out, “the body as a memorial container…furnishes an unmediated access to the remembered past: unmediated, that is, beyond its own withness” (p. 178). In contrast to affective directedness, therefore, body memory is immediate and understood as being in “direct possession of the past” (Merleau-Ponty 2012 [1945], p. 265). Furthermore, given that affective states can themselves be pre-reflectively embodied (Bortolan 2020), negative emotions towards a triggering object might in fact themselves be understood as partly constituted by the non-conceptual body memory that discloses them.

The notion of body memory has various theoretical and clinical implications, not least in resolving some of the conceptual complexity of posttraumatic amnesia. Survivors of complex trauma often face difficulties in being believed, in part due to practices such as victim-blaming (Wilson et al. 2022), but also in regards to their descriptions of memory blanks being taken as confusing or contradictory. This has numerous ethical implications, such as concerning perceptions of deservedness of treatment or criminal justice (Adams-Clark and Freyd 2020; van der Hart and Nijenhuis 1999). Survivors describe feeling invalidated and unheard within healthcare or criminal justice settings, and, understandably, this reinforcement or silencing of one’s traumatic past can be profoundly retraumatising (Aves 2024). Distinguishing between forms of memory—conceptual and non-conceptual, or episodic, semantic and body memory respectively—and identifying the way in which dissociative phenomena can both arise peri-traumatically *and* tacitly thereafter, helps shed light on the multiplicity of survivors’ accounts. Indeed, on this theoretical interpretation, survivors’ varying claims around memory loss can all be better understood. An individual’s sense that something bad has happened despite not knowing what is rendered as legitimate a position as one in which episodic recall is firmly intact.

Although CPTSD and related disorders are not the only circumstances in which memory blanks might conceivably be understood as troubling, the phenomenon may be central among survivors of trauma who dissociate. Indeed, it may turn out to be an identifying feature of conditions associated with complex trauma. Given that dissociation and amnesia are not explicitly assessed for in key diagnostic tools such as the ITQ (Cloitre et al. 2018), this highlights the need for clinicians to investigate these in other ways. For although struggling with gaps in trauma memory is less visibly apparent than symptoms such as flashbacks and nightmares, it can be an equal source of difficulty. Survivors of complex trauma ought to be supported not only in regards to what they can remember, but what they feel they cannot.

## Footnotes

1 It is recognised that not all individuals who have experienced traumatic events endorse the term ‘survivor’. Some feel that ‘victim’ best suits their experience, while others prefer to describe the event or ensuing psychiatric diagnosis. Participants in the present study did not explicitly self-identify as survivors; however, as ‘survivor’ has gained momentum within trauma research and clinical practice, and is generally considered to emphasise agency and recovery, it is the term used within this article.

2 An exploration of how episodic memory is diminished in trauma is beyond the scope of this article. Those interested in the effects of trauma upon episodic memory (including physiological explanatory models) should turn to a recent meta-analysis conducted by Petzold and Bunzeck (2022). Notable theories include those of Foa and Rothbaum (1998), who propose that the emotional intensity of traumatising events disrupt the cognitive processes of attention and memory at the time of encoding, and Ehlers and Clark (2000), who propose trauma memory is poorly integrated into the database of autobiographical knowledge as a result of difficulty in conceptually processing the events.

3 This is not to say that sociocultural dimensions of taboo, which may relate to specific kinds of traumatic events (e.g. incest) or be particularly strongly present in certain cultural contexts (e.g. traumatic loss following a family member’s suicide in some Asian populations) are unrelated to posttraumatic amnesia. Phenomenologically, such taboos may play a constitutive role in how the self experiences, remembers, or fails to remember, a particular event. These dynamics are beyond the scope of this paper, however. The point I wish to make here is that there is a difference in character between a painfully shameful *thought* about a past trauma, and a *body memory* in which traumatic duress both springs from and impacts upon the body.

4 Husserl states that the living present is an “affective unity” (2001 [1920–1926], p. 216), integrating moments across which particular phenomena exert an affective pull. This affective pull can vary from object to object, “arch[ing] out prominently or becom[ing] more flattened depending on the alterations of the living present…the differences in freshness in which all present objects…altogether, gain (or in the opposing case, lose) something of the vivacity of consciousness, of affective force” (2001 [1920–1926], p. 216). Dissociative body memory may, *in extremis* and as a result of its distancing effects, severely diminish the affective force of presently given objects, with corresponding consequences for recollection. There is, however, a tension that must be borne in mind between Husserl’s strong transcendental account of time consciousness and its empirical variation in psychopathology (see Fernandez 2015).
